# IgG N-Glycosylation During Atorvastatin Therapy After Acute Coronary Syndrome is Associated with LDL Cholesterol Reduction

**DOI:** 10.3390/jcm15083056

**Published:** 2026-04-16

**Authors:** Domagoj Mišković, Nikol Mraz, Barbara Radovani Trbojević, Ivana Jurin, Ana Đanić Hadžibegović, Ivan Gudelj, Gordan Lauc, Irzal Hadžibegović

**Affiliations:** 1 General Hospital “Dr. Josip Benčević” Slavonski Brod, 35000 Slavonski Brod, Croatia; domagoj1304@gmail.com; 2 Faculty of Dental Medicine and Health, Josip Juraj Strossmayer University of Osijek, 31000 Osijek, Croatia; ana_djanic@yahoo.com; 3 Genos Glycoscience Research Laboratory, 10000 Zagreb, Croatia; nmraz@genos.hr (N.M.); barbara.radovani@uniri.hr (B.R.T.); glauc@genos.hr (G.L.); 4 Clinic for Cardiovascular Medicine, Dubrava University Hospital, 10000 Zagreb, Croatia; ivanajurin1912@gmail.com; 5 University Hospital Centre Zagreb, 10000 Zagreb, Croatia; 6 School of Medicine, University of Zagreb, 10000 Zagreb, Croatia; 7 Faculty of Pharmacy and Biochemistry, University of Zagreb, 10000 Zagreb, Croatia; ivan.gudelj@uniri.hr

**Keywords:** acute coronary syndrome, statin therapy, lipid management, percutaneous coronary intervention, N-glycosylation, immunoglobulin G

## Abstract

**Background/Objective**: Immunoglobulin G (IgG) N-glycosylation is an important regulator of immune function and systemic inflammation and has been associated with cardiometabolic diseases. However, little is known about how IgG glycosylation changes during the course of acute coronary syndrome (ACS) and whether these alterations relate to lipid-lowering response after the initiation of statin therapy. The primary aim of this study was to investigate IgG N-glycosylation following ACS and evaluate its association with response to atorvastatin therapy defined as baseline LDL cholesterol reduction of ≥50%. **Methods**: In this prospective cohort study, 79 statin-naïve patients hospitalized for the first episode of ACS and treated with atorvastatin 80 mg daily after percutaneous coronary intervention were followed longitudinally. Plasma samples were collected at admission (acute phase), discharge (subacute phase), and follow-up (chronic phase). A control group of 21 individuals received atorvastatin for primary prevention. IgG was isolated from plasma, and N-glycans were released, fluorescently labeled with 2-aminobenzamide, and analyzed using hydrophilic interaction-based ultra-high-performance liquid chromatography with fluorescence detection. Derived glycan traits were calculated, including agalactosylated (G0), monogalactosylated (G1), digalactosylated (G2), core fucosylated (F), bisected (B), and sialylated (S) glycans. **Results**: No significant differences in derived IgG glycan traits were observed between ACS patients and controls at baseline or follow-up. Within the ACS group, a longitudinal analysis revealed significant increases in G0 and F and a decrease in G2 between the acute and chronic phases. A total of 65% of patients achieved ≥50% reduction in LDL cholesterol (LDL-C), whereas only 22% reached the guideline-recommended LDL-C target of <1.4 mmol/L. Patients achieving ≥50% LDL-C reduction exhibited consistently higher G0 and lower G2 and S across disease phases. In a subgroup of patients with baseline LDL-C >3.9 mmol/L, those who failed to achieve ≥50% LDL-C reduction had significantly lower G0 and higher S across all time points. **Conclusions**: Specific glycan traits are associated with the degree of LDL-C reduction achieved during statin therapy, particularly in patients with high baseline LDL-C. These findings suggest that IgG glycosylation patterns may reflect biological phenotypes associated with differential lipid-lowering responsiveness after ACS.

## 1. Introduction

Cardiovascular disease (CVD) is still the most common cause of mortality and morbidity worldwide, with a major impact on the healthcare system and the socioeconomic status of the population [1,2]. Acute coronary syndrome (ACS) is frequently the first clinical manifestation of CVD and encompasses unstable angina (UA), non-ST-segment elevation myocardial infarction (NSTEMI), and ST-segment elevation myocardial infarction (STEMI). The underlying pathological substrate of ACS is atherosclerosis, a chronic inflammatory disease of the arterial wall characterized by lipid accumulation, immune cell infiltration, and vascular remodeling. Plaque rupture, plaque erosion, and the presence of calcified nodules are the principal mechanisms leading to ACS events [3].

In addition to revascularization, lipid-lowering therapy (LLT) is a fundamental component of secondary prevention after ACS. High-intensity statins, particularly atorvastatin and rosuvastatin, represent first-line therapy and typically reduce low-density lipoprotein cholesterol (LDL-C) levels by approximately 50% from baseline values [4,5,6]. Numerous clinical trials have demonstrated that lower achieved LDL-C levels after ACS are associated with a reduced risk of recurrent cardiovascular events [7]. Beyond absolute LDL-C levels, the cumulative exposure to elevated LDL-C over time also plays a critical role in cardiovascular risk. Individuals with the highest cumulative exposure to LDL-C before the age of 40 have been shown to have approximately a fourfold higher cardiovascular risk later in life compared with those with the lowest exposure [8]. Patients with ACS are therefore considered to be at a very high cardiovascular risk, and the current European Society of Cardiology guidelines recommend achieving LDL-C levels below 1.4 mmol/L, along with at least a 50% reduction from baseline values [1]. Despite the availability of potent lipid-lowering therapies, a substantial proportion of patients do not achieve recommended LDL-C targets in clinical practice. Contemporary strategies for further LDL-C reduction include the addition of ezetimibe, proprotein convertase subtilisin/kexin type 9 inhibitors (PCSK9i), inclisiran, or bempedoic acid. Nevertheless, real-world studies consistently demonstrate suboptimal LDL-C control. The EUROASPIRE V survey, which included more than 7000 patients from 27 countries, reported that fewer than one-third of patients with coronary artery disease achieved LDL-C levels below 1.8 mmol/L [9]. Similarly, a Croatian single-center registry study showed that only 33% of patients achieved LDL-C <1.4 mmol/L or ≥50% LDL-C reduction 1 year after ACS [10]. Potential explanations include suboptimal statin adherence, concerns about adverse effects, insufficient statin dosing, and therapeutic inertia in intensifying lipid-lowering therapy [11]. Identifying biological factors associated with variability in lipid-lowering response could therefore help improve risk stratification and treatment optimization.

Glycosylation, which is the covalent attachment of glycans to proteins or lipids, is one of the most complex and variable post-translational modifications in humans. Glycans play an essential role in numerous biological processes, including protein folding, cell signaling, and immune regulation. Given the large number of enzymes involved in glycan biosynthesis and modification, glycosylation patterns are highly sensitive to physiological and pathological changes. Alterations in glycosylation have therefore attracted increasing interest as potential biomarkers in a wide range of diseases, including cardiovascular disorders [12]. Among the different types of glycosylation, N-glycosylation, where glycans are attached to the nitrogen atom of the asparagine side chain, has been extensively studied in the context of inflammation and cardiometabolic disease. Dysregulation of N-glycosylation has been implicated in endothelial dysfunction, leukocyte recruitment, and vascular inflammation, all of which contribute to the development and progression of atherosclerosis [13,14,15]. Glycosylation of adhesion molecules and endothelial proteins can influence leukocyte adhesion and migration across the vascular wall, thereby modulating inflammatory responses in the atherosclerotic plaque [16,17].

IgG glycosylation patterns are influenced by multiple factors, including age, sex, lifestyle, and metabolic status. Previous studies have demonstrated associations between IgG N-glycosylation and lipid metabolism, as well as dyslipidemia [18]. Additionally, alterations in glycosylation of lipoproteins and apolipoproteins, particularly changes in sialylation, have been linked to lipid metabolism and atherosclerotic processes [19]. A population-based study also demonstrated associations between plasma protein N-glycome composition and postprandial lipemic responses, further supporting the interaction between glycosylation and lipid metabolism [20]. Experimental studies investigating macrophage lipid metabolism have similarly suggested that glycan composition of lipoproteins can influence their biological functions and atherogenic potential [21].

IgG N-glycosylation is a key regulator of antibody effector functions and systemic immune responses. Each IgG molecule contains a conserved N-glycosylation site in the Fc region, and variations in glycan composition can substantially alter interactions with Fc receptors and complement components. Therefore, structural changes, such as galactosylation, fucosylation, bisection, and sialylation, influence the balance between pro- and anti-inflammatory immune activity [19,20,21]. Given the complex interplay between inflammation, immune regulation, and lipid metabolism in atherosclerosis, alterations in IgG N-glycosylation may provide insight into biological mechanisms underlying cardiovascular disease and treatment response. However, little is known about how IgG glycosylation changes during the course of ACS or whether these changes are associated with the response to lipid-lowering therapy.

Therefore, the primary aim of the present study was to investigate the association between IgG N-glycosylation and lipid-lowering response following ACS. Specifically, we sought to determine whether IgG glycan profiles differ significantly between ACS patients who achieved ≥50% LDL-C reduction during maximum dose atorvastatin therapy after percutaneous coronary intervention (PCI) and those who did not. Our secondary aims included the analysis of significant differences between ACS patients and controls and also whether IgG glycosylation changes significantly during the course and specific treatment of ACS.

## 2. Patients and Methods

### 2.1. Participants

This prospective cohort study was conducted at the General Hospital “Dr. Josip Benčević” in Slavonski Brod, Croatia, within the Department of Internal Medicine, Division of Cardiology, between September 2022 and April 2025. The study protocol was approved by the Ethics Committee of the Faculty of Medicine, Josip Juraj Strossmayer University of Osijek, and by the Ethics Committee of the General Hospital “Dr. Josip Benčević”. All participants provided written informed consent prior to enrollment. A total of 100 participants were included and divided into a study group and a control group.

Sample size calculation for differences between two independent groups and differences between two repeated measurements accounting for a 10% difference in derived glycan traits between groups, with alpha-error of 0.05 and power of 0.8, showed required sample of 19 patients per group. After correcting for expected 50% rate of patients achieving 50% reduction in LDL-C and 25% of patients achieving LDL-C goal of 1.4 mmol/L among ACS patients, the required number of patients for subanalyses in the ACS groups was calculated at 76. The study group consisted of 79 statin-naïve patients hospitalized for a first episode of acute coronary syndrome (ACS) without previously diagnosed coronary artery disease or peripheral arterial disease. Among these patients, 62 (78%) were male, with a median age of 58 years (range 36–74 years). The diagnosis of ACS was established according to the European Society of Cardiology guidelines based on clinical presentation, electrocardiographic changes, and laboratory findings. Inclusion required ischemic chest pain at rest or an equivalent ischemic symptom lasting longer than 10 min within 12 h of first medical contact, accompanied by at least one additional diagnostic criterion, including significant ST-segment changes or elevated cardiac troponin I levels.

All ACS patients underwent early invasive coronary angiography and PCI of the culprit coronary lesion(s) within 24 h of admission. High-intensity lipid-lowering therapy with atorvastatin 80 mg daily was initiated in all patients, who continued taking regularly the same dose for at least 1 year. Demographic, clinical, and laboratory data were collected, including information on coronary revascularization and culprit coronary artery. Patients transferred from non-PCI centers were excluded to ensure standardized follow-up.

Exclusion criteria were defined to minimize the influence of other pathological conditions that could affect IgG glycosylation patterns, and they are presented in Table 1. After accounting for all inclusion and exclusion criteria, from a total number of 542 ACS patients receiving coronary angiography and PCI within 24 h of admission in the center, 79 (15%) were included in this study.

The control group consisted of 21 individuals without a history or symptoms of coronary artery disease. Among controls, 11 (52%) were male, with a median age of 62 years (range 46–72 years). Statin therapy was initiated for the first time in these individuals as part of primary prevention due to elevated lipid levels (total cholesterol >7 mmol/L and LDL-C >3 mmol/L). All control participants underwent exercise stress testing and transthoracic echocardiography. Individuals with a positive stress test, reduced left ventricular ejection fraction, or regional wall motion abnormalities were excluded. In the control group, atorvastatin was initiated at a daily dose of 20 mg. the differences in demographic and clinical characteristics of ACS patients and controls are shown in Table 2.

### 2.2. Methods

#### 2.2.1. Plasma Sampling

Blood samples were collected at predefined time points. In patients with acute coronary syndrome (ACS), blood was obtained at three time points: at baseline during hospital admission and prior to the initiation of ACS therapy (acute phase), at hospital discharge (subacute phase; median 5 days after baseline; range: 3 to 7 days), and during outpatient follow-up (chronic phase; median 62 days after baseline, range: 57 to 68 days). In the control group, blood samples were collected at two time points: at baseline before the initiation of statin therapy and at follow-up, with a median of 64 days after baseline. Plasma was separated from whole blood and stored until further glycomic analysis whereas LDL-C concentrations were measured using the same institutional protocol. All patients included in the study underwent all planned plasma samplings, and no data derived from samplings were missing. Primary lipid management measure used for analyses was ≥50% reduction in baseline LDL-C at the follow-up sampling. Guidelines recommended achievement of a target value <1.4 mmol/L was used as a secondary lipid management measure.

#### 2.2.2. Isolation of IgG from Human Plasma

Immunoglobulin G (IgG) was isolated from human plasma samples using CIM^®^ r-Protein G LLD 0.05 mL monolithic 96-well plates (2 µm channels; BIA Separations, Ajdovščina, Slovenia; Cat. No. 120.1012-2), following previously described protocols [22]. To minimize batch effects, plasma samples were randomly distributed across multiple 96-well plates containing study samples, standards, and blanks. Prior to IgG isolation, plasma samples were filtered through AcroPrep™ Advance 1 mL 0.45 μm 96-well filter plates (Pall Corporation, New York, NY, USA) and diluted in phosphate-buffered saline (PBS). The monolithic plates were preconditioned according to the manufacturer’s instructions. Filtered plasma samples were then applied to the plate to allow IgG binding to immobilized protein G. After binding, the plate was washed with PBS to remove unbound proteins and impurities. Bound IgG was eluted under acidic conditions using 0.1 M formic acid (pH 2.5) and immediately neutralized with 1 M ammonium bicarbonate (Acros Organics, Fair Lawn, NJ, USA). Eluted fractions were collected into a 1 mL collection plate and dried using vacuum centrifugation. Dried IgG samples were stored at −20 °C until further processing.

#### 2.2.3. N-Glycan Release, Labeling, and Purification

For IgG denaturation, 30 µL of 1.33% sodium dodecyl sulfate (SDS) (Sigma-Aldrich, St. Louis, MO, USA) was added to each sample, followed by incubation at 65 °C for 10 min. Denaturation was terminated by adding 10 µL of 4% Igepal CA-630 (Sigma-Aldrich, St. Louis, MO, USA), and samples were gently mixed for 15 min. N-glycans were enzymatically released through incubation with PNGase F (1.2 U; Promega, Madison, WI, USA) at 37 °C for 18 h. Released N-glycans were fluorescently labeled using 2-aminobenzamide (2-AB) in the presence of 2-picoline borane in a reaction mixture containing 30% acetic acid in dimethyl sulfoxide (DMSO). The labeling reaction was performed at 65 °C for 2 h. Labeled glycans were purified using solid-phase extraction (SPE) on AcroPrep™ wwPTFE 0.2 μm filter plates (Pall Corporation, Port Washington, NY, USA). The plates were preconditioned according to the manufacturer’s instructions. The samples were mixed with 700 µL of cold 96% acetonitrile (ACN) and applied to the SPE plate. Glycans were washed five times with 100% ACN to remove excess labeling reagents and contaminants. Purified glycans were eluted in two sequential steps using 90 µL of ultrapure water, followed by centrifugation at 1000× *g* for 5 min. Purified glycan samples were subsequently stored at −20 °C until chromatographic analysis.

#### 2.2.4. HILIC-UHPLC-FLR Analysis of IgG N-Glycans

Fluorescently labeled IgG N-glycans were analyzed using hydrophilic interaction ultra-high-performance liquid chromatography with fluorescence detection (HILIC-UHPLC-FLR) on an Acquity UPLC H-Class system (Waters, Milford, MA, USA), following previously described protocols with minor modifications to optimize separation [23]. The fluorescence detector was set to an excitation wavelength of 250 nm and an emission wavelength of 428 nm. Chromatographic separation was performed on a Waters UPLC Glycan BEH amide column (130 Å, 1.7 μm particles, 2.1 × 100 mm), maintained at 60 °C, while the samples were held at 10 °C prior to injection. The mobile phase consisted of 100 mM ammonium formate in water (pH 4.4), as solvent A, and acetonitrile (ACN), as solvent B. A linear gradient from 25% to 38% solvent A was applied over 29 min at a flow rate of 0.40 mL/min. Chromatographic data were initially processed using the instrument’s software, followed by manual adjustment of integration boundaries to ensure consistent peak assignment across samples. Chromatograms were divided into 24 glycan peaks (GP1–GP24), corresponding to previously characterized IgG N-glycan structures validated by LC–MS analysis, according to published annotations [23].

The derived glycan traits were calculated from the directly measured glycan peaks to summarize major glycosylation features, including agalactosylated glycans (G0), monogalactosylated glycans (G1), digalactosylated glycans (G2), core fucosylated glycans (F), glycans containing bisecting N-acetylglucosamine (B), and sialylated glycans (S).

#### 2.2.5. Statistical Analysis

Categorical variables were presented as proportions and tested with the chi-square test to assess the differences between groups. The Shapiro–Wilk test was used to assess the normality of the distributions of all continuous variables. All numerical variables showed non-normal distributions and were, therefore, presented as medians with full ranges (minimum and maximum values). The differences in continuous variables between the observed groups were tested using the Mann–Whitney test for two independent samples. The differences between repeated measurements of continuous variables in the same subjects at different time intervals were tested with the Wilcoxon signed-rank test. The differences in N-glycosylation were measured between the patients and controls at the initial and follow-up measurements in order to determine the differences in the impact of acute coronary syndrome and the introduction of statin therapy on glycosylation over the observed time.

The correlations between continuous numerical values were tested using the Spearman correlation test. The level of association of the relevant variables investigated with LDL-C reduction within different groups was analyzed via logistic regression. The significance level was set at *p* < 0.05. The statistical package SPSS for Windows 11.0.3 was used for the statistical analysis (SPSS Inc., Chicago, IL, USA).

## 3. Results

### 3.1. Comparison of IgG N-Glycan-Derived Traits Between ACS Patients and Controls

**Figure 1:**
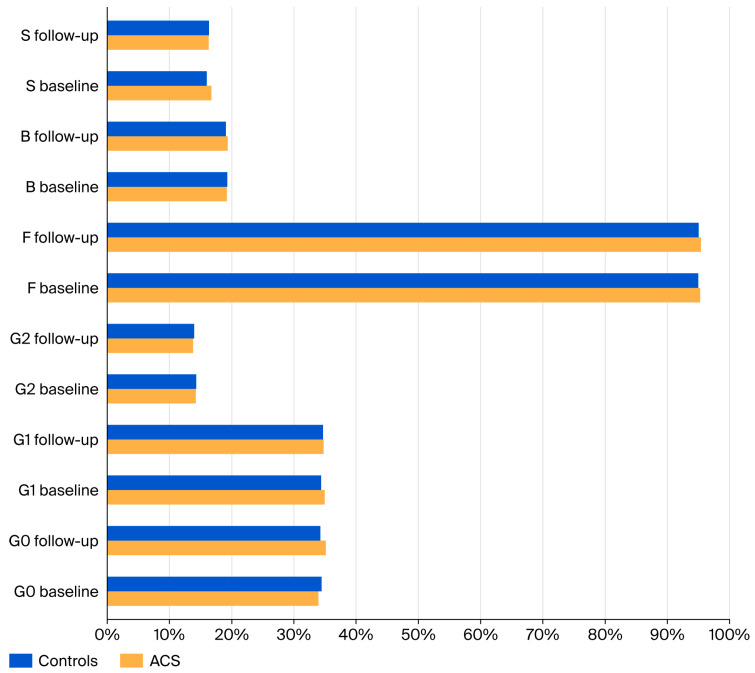
Comparison of derived IgG N-glycan traits between ACS patients and controls at baseline and follow-up plasma sampling.

The comparison of IgG N-glycan-derived traits between the patients with acute coronary syndrome (ACS) and controls showed no statistically significant differences in the percentages of agalactosylated (G0), monogalactosylated (G1), digalactosylated (G2), fucosylated (F), bisected (B), or sialylated (S) glycans at either baseline or follow-up measurements (Figure 1).

### 3.2. Longitudinal Changes in IgG N-Glycosylation in ACS Patients

The longitudinal analysis within the ACS group demonstrated significant changes in IgG N-glycosylation during the course of the disease. A significant increase in the proportion of agalactosylated glycans (G0) was observed between baseline and follow-up (Wilcoxon signed-rank test, *p* = 0.030). In addition, the proportion of core fucosylated glycans (F) significantly increased during the same period (Wilcoxon signed-rank test, *p* < 0.001). Conversely, the proportion of digalactosylated glycans (G2) significantly decreased between baseline and follow-up (Wilcoxon signed-rank test, *p* = 0.010) (Figure 1).

### 3.3. Impact of LDL-Lowering Targets

Among ACS patients, 51 individuals (65%) achieved an LDL cholesterol (LDL-C) reduction of at least 50% compared with baseline values. In contrast, only 17 patients (22%) achieved the guideline-recommended LDL-C target of <1.4 mmol/L at the last follow-up visit.

There were no significant differences in demographic or clinical characteristics between patients who achieved ≥50% LDL-C reduction and those who did not, except for significantly higher baseline LDL-C and HDL-C concentrations in patients who achieved ≥50% LDL-C reduction (Table 3). No significant differences in demographic or clinical characteristics were observed with respect to the attainment of the LDL-C target of <1.4 mmol/L.

**Figure 2:**
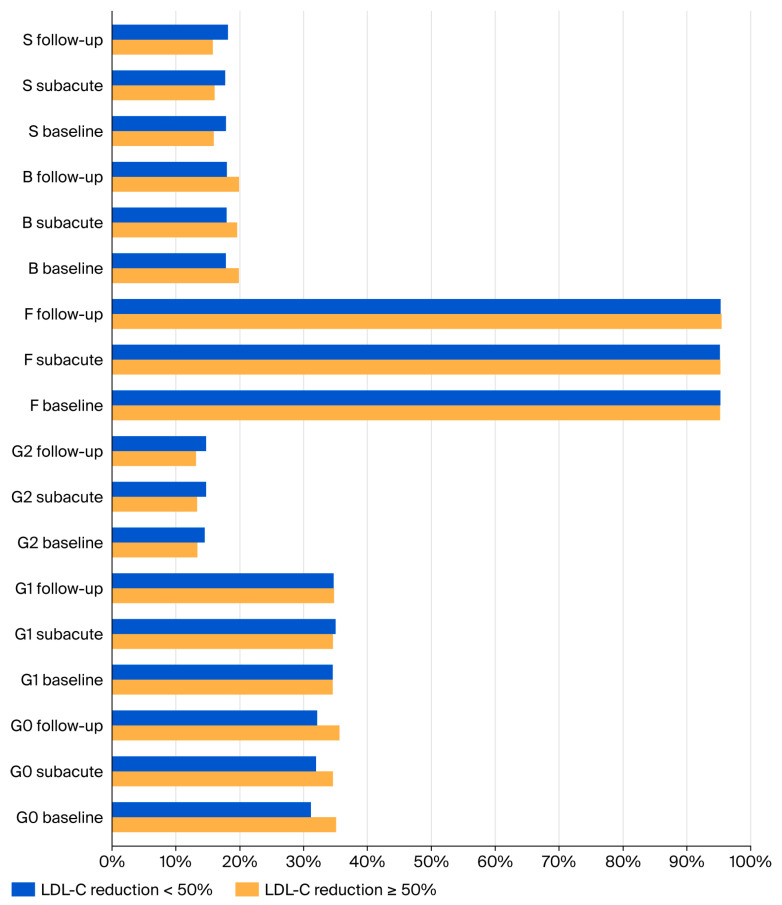
Comparison of derived IgG N-glycan traits between ACS patients who achieved ≥50% LDL-C reduction and those who did not across baseline, subacute, and follow-up sampling.

With regard to IgG glycosylation, the patients who achieved ≥50% LDL-C reduction exhibited consistently higher percentages of agalactosylated glycans (G0) and lower percentages of digalactosylated (G2) and sialylated (S) glycans across all disease phases. In addition, higher levels of bisected glycans (B) were observed in the subacute and chronic phases in patients achieving ≥50% LDL-C reduction (Figure 2).

In contrast, no statistically significant differences in IgG N-glycan traits were observed between patients who achieved LDL-C <1.4 mmol/L and those who did not.

### 3.4. Correlations with Clinical and Laboratory Parameters

Age showed a positive correlation with agalactosylated glycans (G0) (Spearman’s rho = 0.288, *p* = 0.010) and bisected glycans (B) (rho = 0.280, *p* = 0.013), and it showed a negative correlation with monogalactosylated glycans (G1) (rho = −0.234, *p* = 0.038) and digalactosylated glycans (G2) (rho = −0.347, *p* = 0.002).

Baseline LDL-C concentration was positively correlated with G0 (rho = 0.250, *p* = 0.026) and negatively correlated with sialylated glycans (S) (rho = −0.359, *p* = 0.001) in the acute phase.

No other significant correlations were observed between the derived glycan traits and clinical characteristics, including sex, smoking status, diabetes mellitus, or arterial hypertension.

In the logistic regression analysis adjusting for age and baseline LDL-C levels, glycan traits G0, G1, B, and S were not independently associated with ≥50% LDL-C reduction achievement.

### 3.5. Subanalysis of Patients with Baseline LDL-C Higher than Median Value

Because baseline LDL-C levels showed significant correlations—both agalactosylation (G0) and sialylation (S) and patients with ≥50% LDL-C reduction had significantly higher baseline LDL-C levels—an additional subanalysis was performed in patients with baseline LDL-C values above 3.9 mmol/L, corresponding to levels higher than median baseline LDL-C level in the ACS cohort.

**Figure 3:**
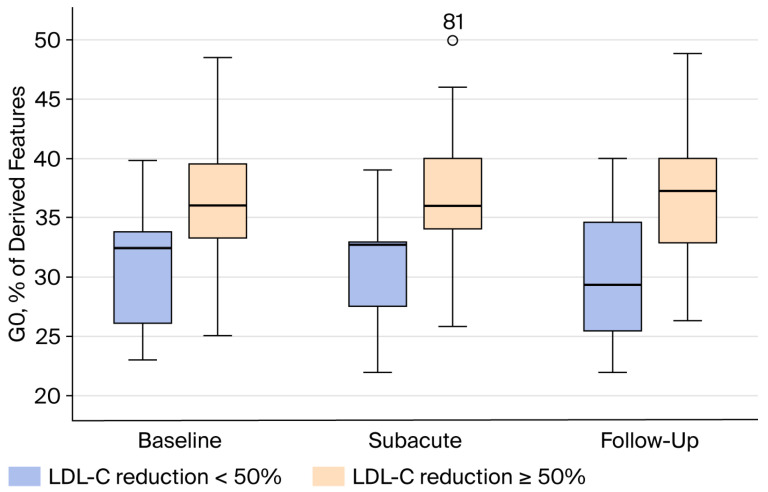
Differences in agalactosylated (G0) IgG N-glycan traits among ACS patients with baseline LDL-C ≥3.9 mmol/L according to achievement of ≥50% LDL-C reduction at baseline, subacute, and follow-up sampling.

**Figure 4:**
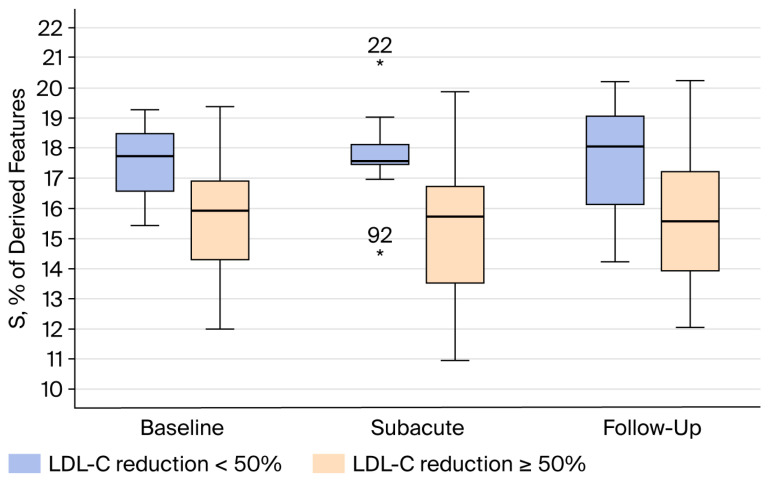
Differences in sialylated (S) IgG N-glycan traits among ACS patients with baseline LDL-C ≥3.9 mmol/L according to achievement of ≥50% LDL-C reduction at baseline, subacute, and follow-up sampling.

Among the 42 patients (53%) with baseline LDL-C >3.9 mmol/L, those who did not achieve ≥50% LDL-C reduction during follow-up exhibited significantly lower levels of agalactosylated glycans (G0) and higher levels of sialylated glycans (S) across all three disease phases compared with patients who achieved the target reduction (Figure 3 and Figure 4).

## 4. Discussion

In this prospective cohort study of statin-naïve patients presenting with a first episode of acute coronary syndrome (ACS) and treated with high-intensity atorvastatin following percutaneous coronary intervention, we observed measurable longitudinal changes in IgG N-glycosylation during the course of the disease. Specifically, significant increases in agalactosylated and core fucosylated glycans and a decrease in digalactosylated glycans were observed between the acute and chronic phases. In addition, specific IgG glycan traits were associated with the degree of LDL cholesterol (LDL-C) reduction achieved during follow-up. The patients who achieved ≥50% LDL-C reduction showed consistently higher agalactosylation and lower digalactosylation and sialylation across disease phases. Importantly, in the subgroup of patients with very high baseline LDL-C (>3.9 mmol/L), failure to achieve ≥50% LDL-C reduction was consistently associated with lower agalactosylation and higher sialylation across all sampling time points. Reduced galactosylation of IgG has been consistently associated with aging and chronic inflammatory conditions, whereas increased sialylation has been linked to altered immune regulation and anti-inflammatory signaling [24,25].

Accumulating evidence indicates that IgG glycosylation is altered in cardiometabolic diseases, including coronary artery disease (CAD) and dyslipidemia. In a cross-sectional study of individuals with angiographically confirmed CAD, significant differences in IgG N-glycan composition were observed compared with subjects with normal coronary arteries [26]. Notably, sialylated IgG N-glycan structures were negatively associated with the presence of CAD, suggesting a potential protective or anti-inflammatory role for IgG sialylation in atherosclerotic disease. In addition, alterations in galactosylation and the presence of bisecting N-acetylglucosamine have been linked to pro-inflammatory IgG effector functions and enhanced immune activation in cardiovascular pathology. These findings support the concept that IgG glycosylation patterns reflect systemic inflammatory processes relevant to atherosclerosis and cardiovascular risk. Consistent with this notion, altered IgG glycosylation has also been reported in dyslipidemia and other metabolic disorders, further highlighting the interplay between immune regulation and lipid metabolism [27]. Our findings extend these observations by demonstrating that IgG glycosylation may also undergo dynamic remodeling following ACS and may be associated with the lipid-lowering response during statin therapy.

Low-density lipoproteins play a central causal role in the development of atherosclerotic cardiovascular disease, supported by genetic, epidemiologic, and clinical evidence [7]. An intensive reduction in LDL-C after ACS substantially lowers the risk of recurrent cardiovascular events and is therefore a cornerstone of secondary prevention [28,29]. Nevertheless, variability in response to statin therapy remains clinically relevant, and a considerable proportion of patients fail to achieve recommended lipid targets despite high-intensity therapy.

The relationship between IgG glycosylation and lipid-lowering response observed in our study may reflect the close interaction between immune regulation, inflammation, and lipid metabolism. Atherosclerosis is increasingly recognized as a chronic inflammatory disease in which both innate and adaptive immune pathways contribute to plaque formation and progression. Glycosylation changes in IgG may therefore represent systemic immunometabolic signatures associated with lipid metabolism and cardiovascular risk.

In our cohort, the patients achieving ≥50% LDL-C reduction had consistently higher agalactosylation and lower sialylation compared with those with a weaker lipid-lowering response. Although causality cannot be inferred, this pattern suggests that baseline immune metabolic phenotypes reflected in IgG glycosylation may be associated with differential responsiveness to statin therapy. Interestingly, these associations were observed for relative LDL-C reduction but not for attainment of the absolute LDL-C target of <1.4 mmol/L.

The current European Society of Cardiology guidelines recommend both ≥50% LDL-C reduction and strict absolute LDL-C targets in patients with ACS [1]. H owever, absolute LDL-C levels are strongly influenced by baseline concentrations, treatment adherence, and subsequent therapy intensification. In contrast, relative LDL-C reduction may reflect the intrinsic biological response to statin therapy better. The observed association between IgG glycan traits and relative LDL-C reduction therefore suggests that glycosylation patterns may capture underlying biological responsiveness rather than treatment intensity.

The most clinically relevant observation of our study concerns patients with baseline LDL-C of >3.9 mmol/L (higher than median LDL-C at baseline). This subgroup analysis was carried out because G0 and S showed significant correlation with higher baseline LDL-C, which was also linked with adequate statin response. In this subgroup, lower agalactosylation and higher sialylation consistently identified patients who failed to achieve ≥50% LDL-C reduction, and this distinction was present across all disease phases. The persistence of this pattern suggests that IgG glycosylation may represent a relatively stable biological phenotype rather than a transient response to acute inflammation during ACS. If confirmed in larger studies, such glycan signatures could potentially help identify patients who are less likely to achieve sufficient LDL-C reduction with statin monotherapy and who might benefit from earlier intensification of lipid-lowering therapy.

Several strengths of this study should be highlighted. First, the prospective design included homogenous statin-naïve patients experiencing their first ACS event (STEMI or high-risk NSTEMI treated early with PCI of the culprit lesion), minimizing the confounding effects of prior lipid-lowering therapy. Second, serial plasma sampling allowed the evaluation of IgG glycosylation across defined disease phases. Third, IgG glycome profiling was performed using a validated chromatographic approach with a standardized derivation of glycan traits.

However, several limitations should also be considered. The overall sample size, particularly in the subgroup analyses, was modest, potentially limiting statistical power. Due to the strict inclusion and exclusion criteria and limited resources, only 15% of all ACS patients receiving timely PCI in the observed period were included in this study. However, by using strict criteria, we obtained a substantially homogenous ACS group of patients for glycan analyses. The alterations of G0, G2, and F traits between baseline and follow-up measurements in the ACS group must be viewed critically in regard to repeated measurements of seven derived features at three time points, making the statistical conclusions rather weak and only hypothesis-generating. We did not report on median time to first medical contact in the ACS group, which could influence glycan traits at baseline, but we included only patients presenting within 12 h of symptom onset. The control group of patients differed significantly from the ACS group in regard to sex, prevalence of smoking, and baseline LDL-C concentrations, which could potentially be linked with IgG N-glycosylation differences. Also, the atorvastatin dose in the primary prevention was significantly lower, making it an additional potential confounder. However, the control group analyses were used to analyze significant alterations in IgG N-glycosylation linked to ACS itself. We did not prove significant changes between the ACS and control group at baseline and after follow-up. Therefore, we focused our further analyses on lipid management response to maximum statin dose among the ACS group. Also, we only analyzed CRP as an indicator of systemic inflammatory response and found no significant elevations or relation in this homogenous ACS group. Other factors linked to inflammatory vascular response could show different results, and that would be interesting for future studies. Statins were not the only drugs administered to the ACS patients during the course of the disease, and the introduction of other drugs specific to ACS could also influence the results. The ACS patients were homogenous in regard to dual antiplatelet therapy, and bolus doses of heparin during PCI. However, we were not able to identify the potential influence of ACE inhibitors, beta blockers, and diabetes mellitus medication. Observational design precludes causal inference regarding the relationship between IgG N-glycosylation and lipid-lowering response, making our study more hypothesis-generating than conclusive. Furthermore, although IgG glycosylation reflects systemic immune regulation, the biological mechanisms linking glycosylation patterns to lipid metabolism remain incompletely understood. Our study design could not investigate the mechanistic link between IgG N-glycosylation and the pharmacological action of statins—HMG CoA reductase inhibition—and these investigations are warranted since we showed a potential link between specific IgG N-glycan traits and response to statins. Finally, external validation and replication analysis in independent cohorts will be necessary to confirm the reproducibility and clinical relevance of these findings.

In conclusion, IgG N-glycosylation undergoes measurable remodeling during the course of acute coronary syndrome. Specific IgG glycan traits were found to be associated with the degree of LDL-C reduction achieved during statin therapy, particularly among patients with high baseline LDL-C levels and did not change significantly during the course of ACS. These findings, albeit being hypothesis-generating, suggest that IgG glycosylation patterns may reflect biologically distinct lipid response phenotypes and could potentially contribute to improved risk stratification and personalized lipid-lowering strategies in patients with ACS by identifying individuals in need for more aggressive treatment in very early phases of ACS.

## Figures and Tables

**Table 1:** Exclusion criteria.

Exclusion Criteria
Age <18 and >75 years.
Malignant or active autoimmune disease.
History of previous ACS apart from current episode.
Previous statin use.
Inability to take dual antiplatelet therapy regularly and continuously for at least 6 months.
Resistant or secondary hypertension.
Morbid obesity–body mass index (BMI) > 40 kg/m^2^.
Insufficiently well controlled diabetes-glycated hemoglobin (HbA1c) > 8%.
Proven and treated post-traumatic stress disorder.
Treatment with oral contraceptives.
Acute pancreatitis, acute cholecystitis, and acute hepatitis.
Acute systemic inflammation or sepsis at the time of admission.
Continuous heparin use.
Treatment with immunoglobulins for the past 6 months.
Probable or definite familial hypercholesterolemia—Dutch Lipid Clinical Network Score (DLCNS) 6–8.

**Table 2:** Demographic and clinical characteristics of ACS patients and controls.

Clinical Features *N* (%) or Median (Range)	Study Population		*p*-Value
	ACS (*N* = 79)	Controls (*N* = 21)	
Sex, male	62 (78)	11 (52)	0.020 *
Age	58 (36–74)	62 (46–72)	0.288
BMI, kg/m^2^	28 (20–37)	29 (21–34)	0.239
Diabetes	5 (6)	1 (5)	0.630
Smoking	43 (54)	4 (19)	0.003 *
Arterial hypertension	57 (72)	15 (71)	0.578
Creatinine, μmol/L	79 (47–154)	79 (54–123)	0.617
CRP, mg/dL	2.7 (0.4–14.5)	2.4 (0.7–3.2)	0.418
LDL cholesterol, mmol/L, initial	3.9 (2.4–7.6)	4.6 (3.7–6.0)	<0.001 *
HDL cholesterol, mmol/L, initial	1.2 (0.7–1.9)	1.7 (1.1–2.4)	<0.001 *
Triglycerides, mmol/L, initial	1.5 (0.4–4.6)	1.9 (1.2–8)	<0.001 *

* Chi-square test for categorical variables and Mann–Whitney test for continuous variables, presenting statistically significant difference at *p* < 0.05.

**Table 3:** Differences in clinical characteristics of patients with ACS according to whether an initial LDL-C reduction of ≥50% was achieved.

Clinical Features *N* (%) or Median (Range)		Study Population with ACS (*N* = 79)		*p*-Value
		LDL-C Reduction ≥50% *N* = 51 (65%)	LDL-C Reduction <50% *N* = 28 (35%)	
Sex, male		41 (80)	21 (75)	0.388
Age		58 (44–74)	58 (36–74)	0.164
BMI, kg/m^2^		27.3 (19.6–35.5)	29.2 (20–37)	0.232
Diabetes		3 (6)	2 (7)	0.585
Smoking		26 (51)	17 (61)	0.277
Arterial hypertension		39 (77)	18 (64)	0.185
Creatinine, µmol/L		83 (50–154)	76 (40–120)	0.617
CRP, mg/dL		2.1 (1.0–11.6)	3.7 (0.4–14.5)	0.398
LDL cholesterol, mmol/L, initial		4.2 (2.7–7.6)	3.4 (2.4–5.4)	<0.001 *
HDL cholesterol, mmol/L, initial		1.3 (0.9–1.9)	1.1 (0.7–1.9)	0.035 *
Triglycerides, mmol/L, initial		1.6 (0.6–4.6)	1.3 (0.4–3.3)	0.297
Type of ACS	STEMI	36 (71)	22 (79)	0.312
	NSTE–ACS	15 (29)	6 (21)	
Culprit lesion	LAD	18 (35)	8 (29)	0.366
	RCA	19 (37)	9 (32)	
	CxA/OM/RIM	14 (28)	11 (39)	
Left ventricle ejection fraction, %	>50	40 (78)	24 (86)	0.664
	40–50	7 (14)	2 (7)	
	<40	4 (8)	2 (7)	
Number of stents implanted during revascularization		1 (0–3)	1 (0–2)	0.295
P2Y12 medication before revascularization	Prasugrel	34 (67)	12 (43)	0.096
	Ticagrelor	16 (31)	14 (50)	
	Clopidogrel	1 (2)	2 (7)	
Ezetimibe in therapy at discharge or at follow-up		51 (100)	23 (82)	0.004 *

* Chi-square test for categorical variables and Mann–Whitney test for continuous variables, presenting statistically significant difference at *p* < 0.05.

## Data Availability

The data presented in this study are available on request from the corresponding author due to privacy and Ethics Committee restrictions.
